# Effects of feeding drunken horse grass infected with *Epichloë gansuensis* endophyte on animal performance, clinical symptoms and physiological parameters in sheep

**DOI:** 10.1186/s12917-017-1120-6

**Published:** 2017-07-19

**Authors:** Ying Liang, Hucheng Wang, Chunjie Li, Zhibiao Nan, Fadi Li

**Affiliations:** 1 State Key Laboratory of Grassland and Agro-ecosystems, College of Pastoral Agriculture Science and Technology, Lanzhou University, Lanzhou, 730020 China; 2 Second Hospital of Lanzhou University, Lanzhou, 730000 China

**Keywords:** *Achnatherum inebrians*, Biochemistry, Clinical symptoms, *Epichloë* endophyte, Sheep

## Abstract

**Background:**

Many reports showed that grass-endophyte symbiosis induced livestock poisoned. Yet, there is no study evaluating clinical symptoms and physiological parameters in sheep fed *Epichloë gansuensis* endophyte-infected grass. The objective of the present study was to investigate these indexes by feeding sheep with endophyte-infected *A. inebrians* (E+ Group) or endophyte-free *A. inebrians* (E- Group) drunken horse grass or alfalfa hay (Control Group).

**Results:**

The *Epichloë* endophyte caused obvious toxicity symptoms in the sheep fed E+ *A. inebrians*, with 1 of the 5 sheep having died by the 35th day. The feed intake and body weight gain of the E+ Group were significantly less than the E- and control groups (*P <* 0.05). Serum concentrations of alanine aminotransferase (ALT, 45.5 mmol/L) and aspartate aminotransferase for the E+ group (AST, 139.3 mmol/L) were significantly (*P <* 0.05) greater than for the E- (ALT, 31.2 mmol/L; AST, 78.6 mmol/L) and control (ALT, 32.6 mmol/L; AST, 56.6 mmol/L) groups at the fifth week; serum concentration of creatinine for the E+ group (63.8 mmol/L) was also significantly (*P <* 0.05) greater than for E- (56.6 mmol/L) and control groups (58.5 mmol/L). Meanwhile, urine biochemical indices for the E+ group indicated that ketone and occult blood were significantly (*P <* 0.05) elevated compared to the other groups while urine pH values were significantly (*P <* 0.05) acidic. The relative weight of heart, brain, liver, lung and kidney for Group E+ were almost two fold more than the other groups, but uterus weight was about half that found for Group E- or Control.

**Conclusions:**

We conclude that the *Epichloë* endophyte infection is the cause of *A. inebrians* toxicity to sheep. Interestingly, none of the measured parameters differed significantly between E- and the control groups, which implied that drunken horse grass could be utilized efficiently by sheep when not infected by the *Epichloë* endophyte.

## Background

Drunken horse grass (*Achnatherum inebrians*) is distributed mainly within the native grasslands of northern and northwestern China. Unfortunately, once infected by a symbiotic fungal endophytes of *Epichloë*, the grass is a toxic perennial plant and poisonous to grazing animals [1]. On the one hand, grass plants with the endophytic fungus display insect resistance, drought resistance, rapid growth, and strong competitive ability. On the other hand, as noted above, the symbiont produces toxins that cause livestock poisoning, with huge animal productivity losses as a result. Animals fed *A. inebrians* can display symptoms of intoxication, such as sluggishness, tottering, drooping, and glaring [2]. The horse (*Equus caballus*), donkey (*E. asinus*), mule (*E. caballus × E. asinus*), and rabbit (*Oryctolagus cuniculus*) are commonly reported to show symptoms of intoxication, but sheep were not significantly intoxicated when dosed with *A. inebrians* powder [3, 4], which might be the grass without fungal endophytes or rumen fermentation reducing toxicity of endophyte toxins in ruminants but what mechannism is not reported. Previous research has shown that almost 100% of *A. inebrians* plants in natural rangeland are infected by the endophyte *Epichloë gansuensis* [5], and that the main alkaloids produced are ergonovine and ergine [6, 7]. A rabbit-feeding trial comparing the effect of endophyte-infected (E+) and endophyte-free (E-) *A. inebrians*, demonstrated significant clinical intoxication symptoms in animals fed the E+ material [1]. In order to further explore the toxicity status *A. inebrians* when infected by this endophyte, an experiment was carried out on small-tailed Han sheep (*Ovis aries*) fed E+ or E- *A. inebrians.* Resulting clinical symptoms, and physiological or biological effects were observed and analyzed.

## Methods

A feeding experiment of 35 days duration was conducted at the Animal Experimental Station of Gansu Agricultural University, China. All research protocols used in the current experiments were approved by the Animal Ethics Committee of Gansu Province, China.

### Animals used and feeding regime

Drunken horse grass, E+ and E- *A. inebrians* hay, was harvested from the Yuzhong experimental station (N 35°10′, E 103°41′, elevation 1731 m) of Lanzhou University, Gansu, China. To confirm the endophyte infection status, the endophyte was detected by microscopic examination for both E+ and E- plants. Referring to the procedure by Zhang et al. (2011) reported that the alkaloids were determined with Agilent 1100 series high performance liquid chromatography (HPLC) system, ZORBAX - XDB C18 reversed phase chromatographic column, mobile phase flow rate of 1 ml/min, 20 ul, testing with VWD uv monitor [8].

Fifteen 10 to 12 month-old female small-tailed Han sheep were used, and their initial body weights ranged from 17.46 to 22.29 kg. The sheep were randomly divided into 3 groups (E+, E- and control), and each sheep was housed in a metabolic cage (150 cm by 80 cm × 70 cm). Sheep was fed a diet (Table 1) containing E+ or E- *A. inebrians* hay, and the daily dry matter allowance was calculated as 3% of animal body weight. For the control group, alfalfa hay (*Medicago sativa*) replaced *A. inebrians* hay. Animals had free access to water. During a one-week pre-experiment period, and for the five week experiment that followed, the daily diet was divided into three equal parts for feeding to individual animal over the course of the day.

**Table 1:** Ingredient and chemical composition of experimental diets

Item	Group		
	E+	E-	Control
Ingredient, % DM			
Wheat straw	35.00	35.00	35.00
Drunken horse grass hay^a^	25.00	25.00	0.00
Alfalfa hay	0.00	0.00	25.00
Corn	19.70	19.70	19.70
Soybean meal	12.00	12.00	12.00
Wheat bran	7.00	7.00	7.00
Cottonseed oil	1.00	1.00	1.00
NaCl	0.30	0.30	0.30
Nutrient levels^b^, % DM			
ME, MJ/kg DM			
CP	11.71	11.93	13.73
Ca	0.24	0.24	0.52
P	0.26	0.26	0.28
NDF	36.00	36.27	36.67
Alkaloid levels^c^, mg/kg DM			
Ergonovine	178.73	0	0
Ergine	353.34	0	0

^a^Drunken horse grass hay, E+ and E- *A. inebrians* hay, was harvested from the Yuzhong experimental station (N 35°10′, E 103°41′, elevation 1731 m) of Lanzhou University, China

^b^ME was calculated according to Tables of Feed Composition and Nutritive Values in China (2013, 24th ed.); others were measured values

^c^Alkaloid levels were determined with Agilent 1100 series high performance liquid chromatography (HPLC) system, and the alkaloid of E- A. inebrians and alfalfa hay was not detected

During the five-week experiment any clinical symptoms were carefully recorded each day. Feed intake and body weight were measured and samples of blood weekly and urine were collected at the end of 5th week. Sheep heart rates were determined with a medical stethoscope, and rectal temperature also was measured using a mercury thermometer. At the end of the experiment, all animals were weighed, and then slaughtered. The heart, liver, spleen, lung, brain, and uterus of each animal were removed and their respective fresh weights immediately recorded.

### Sampling procedures

Blood samples were drawn from a jugular vein. To obtain samples, an area around the mid and lower third of the jugular vein was shaved and sterilised, pressure applied, and when vascular engorgement occurred, a double ended hypodermic needle inserted, and blood collected into test tubes, and whole blood samples were centrifuged under 3500 rmp for 15 min to obtain serum for the respective chemical determinations. All serum samples were saved - 75 °C freezer until analysed. Glutamic pyruvic transaminase (ALT), aspartate aminotransferase (AST) and creatinine (Cr) were determined by a 7080 automatic biochemistry analyzer (Hitachi, Japan).

About 200-ml urine was collected using a sterile plastic bag while the sheep was urination on the morning of last three days of 5th week. After collection of urine, 0.5 ml of 40% formaldehyde solution per 100 ml of urine was added and sub-samples were stored at −20 °C. Routine urine analyses were carried out using a BW-200 urine analyzer (Yantai, China), including occult blood, urine protein, and ketone levels and leukocyte counts.

At the end of the experiment after slaughter, viscera were removed and major organs including the heart, liver, spleen, lung, kidney, brain, and uterus dissected out, weighed, and the weight expressed as a percentage of body weight, hereafter referred to as the relative weight of organ.

### Statistical analysis

Statistical analyses were performed by a professional statistician using SPSS Version 21.0. Significance was set at *P* < 0.05. For intake, body weight, heart rate, rectal temprature, ralative weight of organ and serum biochemical parameters, these comparisons were made using one-way ANOVA with Tukey multiple comparisons. For the acidic or positive of urine parameters, the ratio was caluclated and significances were anounced using χ^2^ test procedure.

## Results

### **Animal clinical symptoms and performance associated with ingestion of** Achnatherum Inebrians **endophyte**

#### Clinical symptom, feed intake and weight gain

The small-tailed Han sheep used in these feeding experiments appeared to have a severe toxic reaction to E+ *A. inebrians*, including absent-mindedness, blank stares and stumbling, but not to E- *A. inebrians* or the control (Table 2), which indicated that the toxicity of *A. inebrians* was associated with *Epichloë* endophyte infection.

**Table 2:** Effects of ingested *Achatherum inebrians* on clinical symptoms of sheep

Group^1^	Ratio^2^ (%)	Duration (d)	Clinical symptoms
E+	100	Day 1–35	Flagging spirit and bowed-down heads; unsteady gait; unresponsive and anorexia.
	100	Day 1–21; 22–35; 1-35th	Body weight fell sharply during week 1 to 3; and declined gradually thereafter.
			Body temperature rose.
	100	Day 1–14	Heart beat faster at first, but gradually became steady.
	20	At the day 35	By the Day 35, one sheep pupils dilated, neck stiff, limb tics, nasal mucosal bleeding, difficulty breathing, and was dead in about three hours.
E-	100	Day 1–35	Normal and free of all clinical symptoms
	100	Day 1–7	Initially weight declined slightly, and then returned to normal.
Control	100	All 35 days	The physical indicators were normal.

^1^E+ = drunken horse grass, *Epichloë* endophyte-infected; E- = drunken horse grass, endophyte-free

^2^The ratio refers to the animal number of clinical symptoms to total observed objects in the group during the trial period

Additionally, the feed intake of the E+ group was significantly less than E- and control Groups (*P* < 0.05), while the feed intake of E- and control Groups did not differ significantly (*P > 0.05*), with the average feed intake of E+ and E- Groups was reduced by 12.50% and 2.7%, respectively, compared to the Control group during the feeding experiments (Table 3).

**Table 3:** Effects of ingested E+ or E-*Achatherum inebrians* on feed intake and body weight of han sheep^1,2^

Week	Feed intake (g/d)			Body weight (kg)		
	E +	E -	Control	E +	E -	Control
0	434 ± 37.6^b^	488 ± 32.1^a^	510 ± 33.3^a^	20.1 ± 2.3	19.6 ± 4.3	20.9 ± 1.3
1	469 ± 28.9^b^	504 ± 46.3^a^	527 ± 46.3^a^	17.5 ± 1.5	19.2 ± 3.7	21.3 ± 1.0
2	435 ± 50.8^b^	493 ± 32.9^a^	513 ± 38.2^a^	17.3 ± 1.3^b^	20.3 ± 3.2^a^	22.4 ± 1.1^a^
3	458 ± 34.3^b^	507 ± 39.1^a^	529 ± 38.1^a^	15.7 ± 1.1^b^	20.3 ± 3.5^a^	23.1 ± 1.5^a^
4	481 ± 28.1^b^	527 ± 4.3^a^	537 ± 35.7^a^	15.8 ± 1.2^c^	19.4 ± 3.8^b^	22.8 ± 1.3^a^
5	464 ± 31.7^b^	520 ± 49.1^a^	510 ± 39.3^a^	15.0 ± 1.6^b^	20.9 ± 2.6^a^	22.9 ± 0.8^a^

^1^Data were recorded at 3 pm on Saturday of the 0, 1st, 2nd, 3rd, 4th and 5th week

^2^E+ = drunken horse grass, Epichloë endophyte-infected; E- = drunken horse grass, endophyte-free

^a-b^Means within a row among three treatments not bearing a common superscript letter differ (*P ≤ 0.05*)

There was a decline in body weight of the E + group within the first week, while body weights of E- sheep were stable and Control sheep slowly increased. The body weight loss of the E+ group compared to E- and Control groups had become statistically significant after the secend week (*P <* 0.05). Body weight of the E- group was less than that of the Control group, but not significantly so (*P >* 0.05). The average body weight of E+ sheep (15.0 kg) was 28% lighter than E- sheep (20.9 kg) and 34% lighter than Control sheep (22.9 kg) by the end of the feeding experiments (Table 3).

#### Heart rate and rectal temperature

E+ exhibited a transitory elevation of heart rate compared to E- and control sheep in week 2 and week 3 (*P < 0.05*), and after the first two weeks, rectal temperatures of E+ sheep were significantly (*P < 0.05*) greater than the other two groups. However, heart rates and rectal temperatures of E- and Control sheep did not differ significantly (*P > 0.05*) at any time (Table 4).

**Table 4:** Effects of ingested E+ or E- *Achnatherum inebrians* on heart rate and rectal temperatures of han sheep^1, 2^

Week	Heart rate (beats per minute)			Rectal temperature(°C)		
	E +	E -	Control	E +	E -	Control
0	70 ± 4.5	72 ± 3.1	72 ± 3.3	39.1 ± 0.2	39.0 ± 0.2	39.0 ± 0.2
1	78 ± 16.1	74 ± 6.2	70 ± 3.2	39.3 ± 0.3	39.1 ± 0.4	39.1 ± 0.1
2	81 ± 9.4^a^	71 ± 3.9^b^	69 ± 5.2^b^	38.5 ± 0.2^b^	39.0 ± 0.1^a^	39.0 ± 0.1^a^
3	79 ± 3.3^a^	72 ± 3.8^b^	72 ± 3.2^b^	39.8 ± 0.3^a^	39.1 ± 0.2^b^	39.3 ± 0.1^b^
4	73 ± 7.2	68 ± 5.7	67 ± 4.7	39.1 ± 0.2^a^	38.6 ± 0.4^b^	38.8 ± 0.2^b^
5	72 ± 1.5	70 ± 2.1	71 ± 4.9	39.6 ± 0.3^a^	38.7 ± 0.6^b^	38.9 ± 0.3^b^

^1^Data were recorded at 3 pm on Saturday of the 0, 1st, 2nd, 3rd, 4th and 5th week

^2^E+ = drunken horse grass, Epichloë endophyte-infected; E- = drunken horse grass, endophyte-free

^a-b^Means within a row between three treatments not bearing a common superscript letter differ (*P ≤ 0.05*)

#### Urine parameters

Numbers of sheep with acidic urine were significantly (*P <* 0.05) higher for E+ *A. inebrians*-fed sheep than for the E- and control groups. Urine occult blood and ketone levels were also significantly (*P <* 0.05) elevated in E+ sheep, but urine protein and leukocyte levels did not significantly (*P >* 0.05) change (Table 5).

**Table 5:** Effects of ingested E+ or E- *Achnatherum inebrians* on urine parameters of han sheep^a,b,c^

Analysis		Percent of observed values(%)			χ^2^	*P* value
		E+	E-	Control		
pH	< 7.0	50.0	0.0	0.0	16.261	*<* 0.05
	7.0 to 8.0	14.3	14.3	83.3		
	> 8.0	35.7	85.7	16.7		
Occult blood	++	7.7	0.0	0.0	11.505	*<* 0.05
	+	76.9	28.6	30.8		
	−	15.4	71.4	69.2		
Leukocytes	+	7.1	0.0	0.0	1.977	*>*0.05
	−	92.9	100.0	100.0		
Urine protein	++	30.8	21.4	7.7	3.715	*>* 0.05
	+	30.8	57.1	53.8		
	−	38.5	21.4	38.5		
Ketone	+	33.3	14.2	15.4	8.983	*<* 0.05
	−	66.7	85.7	84.6		

^a^Samples were collected at 7:00 am on last three days of 5th week

^b^E+ = drunken horse grass, *Epichloë* endophyte-infected; E- = drunken horse grass, endophyte-free

^c^+ Means positve, ++ seriously positive, − negative

### Serum activity of enzymes and renal function

Ingestion of E+ *A. inebrians* resulted in significantly (*P <* 0.05) elevated aspartate aminotransferase activity (AST) compared to E- and Control groups from week 2 onwards. Alanine aminotransferase (ALT) activity and creatinine levels (Cr) were not significantly elevated in E+ animals during the first 4 weeks (*P > 0.05*), but were increased significantly by the 5th week (*P <* 0.05) (Table 6).

**Table 6:** Effects of ingested E+ or E- *Achatherum inebrians* on selected serum biochemical parameters in han sheep

Items	Group	Weeks					
		0	1	2	3	4	5
Alanine aminotransferase (ALT, mmol/L)	E+	40.0 ± 1.58^B^	40.8 ± 6.18^B^	41.4 ± 10.43^B^	39.8 ± 9.50^B^	44.0 ± 15.64^A^	45.5 ± 9.95^aA^
	E-	39.8 ± 1.30^A^	39.4 ± 1.52^A^	37.2 ± 3.56^A^	31.4 ± 8.62^B^	33.8 ± 8.58^B^	31.2 ± 3.90^bB^
	Control	39.0 ± 1.58^A^	36.4 ± 4.04^B^	39.0 ± 5.0^A^	34.8 ± 7.82^B^	35.6 ± 4.51^B^	32.6 ± 3.58^bB^
Aspartate aminotransferase (AST, mmol/L)	E+	91.6 ± 3.44^C^	98.8 ± 15.89^C^	110.6 ± 22.17^aB^	120.0 ± 34.65^aB^	141.5 ± 51.16^aA^	139.3 ± 40.37^aA^
	E-	90.2 ± 2.17^B^	95.4 ± 4.51^A^	88.8 ± 1.48^bB^	88.2 ± 12.52^bB^	94.0 ± 19.40^bA^	78.6 ± 5.98^bC^
	Control	90.6 ± 2.30^A^	90.8 ± 7.56^A^	84.0 ± 1.73^bB^	91.4 ± 17.60^bA^	95.8 ± 11.67^bA^	86.4 ± 19.05^bB^
Creatinine (Cr, umol/L)	E+	55.7 ± 1.68^D^	78.0 ± 8.63^aA^	69.4 ± 15.96^aB^	60.7 ± 13.58^aC^	64.6 ± 17.34^aC^	63.8 ± 14.79^aC^
	E-	56.7 ± 2.64^B^	70.4 ± 3.65^bA^	60.0 ± 3.54^bB^	59.4 ± 4.28^aB^	56.7 ± 13.39^bB^	56.6 ± 3.65^bB^
	Control	55.3 ± 3.97^A^	55.6 ± 6.23^cA^	55.7 ± 5.03^bA^	49.6 ± 6.27^bB^	57.0 ± 9.03^bA^	58.5 ± 9.35^bA^

^a-c^Means within a column between three treatments not bearing a common superscript letter differ (*P ≤ 0.05*)

^A-D^Means within a line between five periods not bearing a common superscript letter differ (*P ≤ 0.05*)

### *Effects of* Achatherum inebrians *Endophyte on relative weight of organ*

Significant differences in relative weight of organ (*P >* 0.05) were also observed. Ingestion of E+ *A. inebrians* raised heart, brain, liver, lung and kidney relative weight of organ, while its for the uterus was decreased (Table 7).

**Table 7:** Effects of ingested E+ or E- *Achatherum inebrians* on relative weight of organ of han sheep

Treatment	Relative weight of organ						
	Heart	Brain	Liver	Spleen	Lung	Kidney	Uterus
E +	0.366 ± 0.314	0.405 ± 0.347^a^	1.124 ± 0.963^a^	0.092 ± 0.079	1.138 ± 0.977^a^	0.228 ± 0.195^a^	0.036 ± 0.033^b^
E -	0.220 ± 0.310	0.251 ± 0.352^b^	0.633 ± 0.887^b^	0.068 ± 0.096	0.672 ± 0.942^b^	0.125 ± 0.175^b^	0.080 ± 0.112^a^
Control	0.197 ± 0.276	0.209 ± 0.293^b^	0.603 ± 0.845^b^	0.068 ± 0.095	0.618 ± 0.865^b^	0.121 ± 0.169^b^	0.085 ± 0.120^a^

E+ = drunken horse grass, *Epichloë* endophyte-infected; E- = drunken horse grass, endophyte-free

^a-b^Means within a column between three treatments not bearing a common superscript letter differ (*P ≤ 0.05*)

## Discussion

### Clinical symptoms

Feeding experiments demonstrated that the small-tailed Han sheep appeared to have a toxic reaction after ingestion of E+ *A. inebrians*, including absent-mindedness, blank stares and stumbling, but not following ingestion of E- *A. inebrians* or the control feed. At 35-d one small-tailed Han sheep exhibited poisoning symptoms including mydriasis, neck stiffness, limb tic, nasal mucosa bleeding, and weak breathing. It suffered a hypotensive crisis and its condition worsened, with muscle flaccidity, loss of defecation, abdominal swelling, retention of urine, and ultimately death. These symptoms are similar to the toxicity symptoms of ergonovine and ergine [9–11]. Ergonovine poisoning can cause excitement, muscle relaxation and allergic reaction, apparent intelligence depression, gastrointestinal dysfunction and stomach ache. Ergine can cause anesthesia and retroperitoneal fibrosis, urinary tract obstruction and renal failure. Therefore, it was concluded that the sheep fed E+ *A. inebrians* were poisoned by the alkaloids that are produced by the *N. gansuense* endophyte symbiont of *A. inebrians*.

The feed intake of sheep fed E+ *A. inebrians* was significantly less than those fed E- *A. inebrians* or the control group, so the endophytic fungi reduce either palatability or feeding drive of the animals, or possibly both. In the first three days of the experiment, the feed intake reduction of the E+ group was very large but then rose slightly, suggesting initial aversion of the sheep to feed containing endophye infected drunken horse grass, followed by gradual acclimation. This is consistent with prevously published findings [12].

Suppression of feed intake of small tailed han sheep offered endophyte infected *A. inebrians* is consistent with observed body weight loss, so the endophyte infected *A. inebrians* can be considered a factor in poor body condition of small tailed han sheep. This is consistent with a research report of Blaney [13].

Endophyte infected *A. inebrians* fed to sheep resulted in significantly elevated levels of urine ketone bodies and occult blood and increased numbers of animals with acidic urine. Ketone bodies are a product of the decomposition of fatty acids, and include acetoacetate, acetone and ß- hydroxybutyric acid [14–16]. When their production is greater than the liver can metabolize, the ketones will accumulate to produce acidosis. Under physiological conditions, ketone bodies cannot be detected in animal urine, but they can appear with long-term malnutrition, hunger, long-term anesthesia and after a wound. Ergot alkaloids produced by E+ *A. inebrians* have effects on the nervous system, with the increased presence of ketone bodies possibly being responsible for long-term anesthesia, but the specific mechanism of action needs further research.

Occult blood is hemoglobin or red blood cells in urine which cannot be observed by the naked eye directly [17, 18]. The bleeding of different parts of the urinary tract, due to hemolytic disease, poisoning [19] or blood transfusion reactions [20], can cause a positive occult blood reaction. In this study, the poisoning of sheep by feeding E+ *A. inebrians* was very strongly correlated with the positive occult blood reaction, and can be considered a probable cause.

The pH of animal urine is affected by feed properties, and generally herbivore urine is slightly alkaline, but some nutritional metabolic diseases such as ketonemia, or pathogenic heat or malnutrition can make urine acidic [16, 21]. In this study, the observed acidic urine may have been caused by ketone bodies, but the mechanism is still ill-defined.

### Liver and renal function

Serum ALT and AST values are used mainly for the detection of liver disease in livestock, and generally the ALT and AST activity of sheep ranges from 25.0 to 70.0 U/L, 40.0 to 123.0 U/L respectively [22]. In the present study, the activities of serum ALT and AST in E+ sheep were significantly higher than the E- or Control sheep. These results for the E+ group were similar to those prevously published [23–26].

Creatinine is a substance associated with energy metabolism of muscles, and elevation occurs when its discharge is increased. Serum creatinine concentration rises rapidly early in kidney disease [27]. That is consistent with the detected significant increase in the first week of the experiment. The normal range of Serum creatinine is usually less than 150 mmol/L. Reduced creatinine levels are of no clinical significance, but when levels of 250 mmol/L or more are observed, this may inidcate kidney dehydration or heart failure [28].

### Relative weight of organ

E+ *A. inebrians* ingestion brought about higher of brain, liver, lung and kidney, but lower that of the uterus. This result would be expected, given the negative effects in liver and renal function, and more loss of body weight outlined above.

## Conclusions

Ingestion of E+ *A. inebrians* feed by small tailed Han sheep (*O. aries*) resulted in a range of clinical symptoms and biochemical effects. Ingestion of the endophyte not only made serum indices and urine biochemistry abnormal, but also caused absent-mindedness, blank stares and stumbling. The alkaloid secondary metabolites produced by the *Epichloë* endophyte infection of *A. inebrians* are apparently the cause of this toxicity. Interestingly, no significant differences in measured parameters were observed between sheep fed E- *A. Inebrians* and those fed a diet based on alfalfa, which implies that drunken horse grass could be utilized as an animal feed source if free of the *Epichloë* endophyte.

## Abbreviations

ALT: Alanine aminotransferase
AST: Aspartate aminotransferase
Cr: Creatinine
E-: Endophyte-free *A. inebrians*
E+: Endophyte-infected *A. inebrians*
